# Dichlorido(4′-ferrocenyl-2,2′:6′,2′′-terpyridine-κ^3^ *N*,*N*′,*N*′′)zinc acetonitrile monosolvate

**DOI:** 10.1107/S1600536811024950

**Published:** 2011-07-02

**Authors:** Si-Ping Tang, Yong-Lan Feng, Dai-Zhi Kuang

**Affiliations:** a Key Laboratory of Functional Organometallic Materials, Department of Chemistry and Material Science, Hengyang Normal University, Hengyang, Hunan 421008, People’s Republic of China

## Abstract

The title complex, [FeZn(C_5_H_5_)Cl_2_(C_20_H_14_N_3_)]·CH_3_CN, is composed of one Zn^II^ atom, one 4′-ferrocenyl-2,2′:6′,2′′-terpyridine (fctpy) ligand, two Cl atoms and one acetonitrile solvent mol­ecule. The Zn^II^ atom is five-coordinated in a trigonal–bipyramidal geometry by the tridentate chelating fctpy ligand and two Cl atoms.

## Related literature

For 4′-ferrocenyl-2,2′:6′,2"-terpyridine metal complexes, see: Aguado *et al.* (2005 ▶); Constable *et al.* (1994 ▶); Farlow *et al.* (1993 ▶); Tang & Kuang (2007 ▶); Tang *et al.* (2009 ▶).
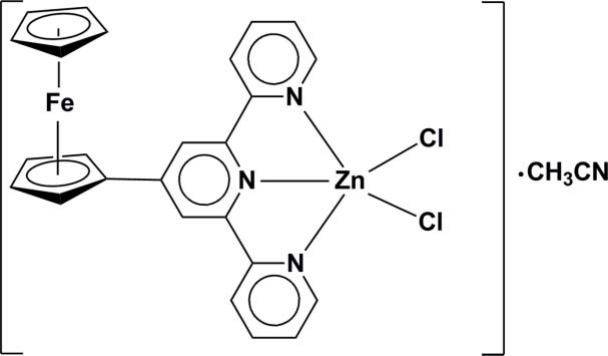


## Experimental

### 

#### Crystal data

[FeZn(C_5_H_5_)Cl_2_(C_20_H_14_N_3_)]·C_2_H_3_N
*M* *_r_* = 594.61
Monoclinic, 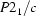

*a* = 13.799 (2) Å
*b* = 12.998 (2) Å
*c* = 14.594 (2) Å
β = 104.514 (4)°
*V* = 2534.0 (6) Å^3^
*Z* = 4
Mo *K*α radiation
μ = 1.75 mm^−1^
*T* = 295 K
0.18 × 0.14 × 0.10 mm

#### Data collection

Bruker APEX area-detector diffractometer
Absorption correction: multi-scan (*SADABS*; Sheldrick, 1996 ▶) *T*_min_ = 0.743, *T*_max_ = 0.844
12737 measured reflections
4938 independent reflections
3301 reflections with *I* > 2σ(*I*)
*R*_int_ = 0.051

#### Refinement

*R*[*F* ^2^ > 2σ(*F* ^2^)] = 0.050
*wR*(*F* ^2^) = 0.131
*S* = 1.02
4938 reflections
317 parameters
H-atom parameters constrained
Δρ_max_ = 0.77 e Å^−3^
Δρ_min_ = −0.40 e Å^−3^

### 

Data collection: *SMART* (Bruker, 2002 ▶); cell refinement: *SAINT* (Bruker, 2002 ▶); data reduction: *SAINT*; program(s) used to solve structure: *SHELXS97* (Sheldrick, 2008 ▶); program(s) used to refine structure: *SHELXL97* (Sheldrick, 2008 ▶); molecular graphics: *SHELXTL* (Sheldrick, 2008 ▶); software used to prepare material for publication: *SHELXTL*.

## Supplementary Material

Crystal structure: contains datablock(s) I, global. DOI: 10.1107/S1600536811024950/jh2301sup1.cif

Structure factors: contains datablock(s) I. DOI: 10.1107/S1600536811024950/jh2301Isup2.hkl

Additional supplementary materials:  crystallographic information; 3D view; checkCIF report

## Figures and Tables

**Fig. 1.**
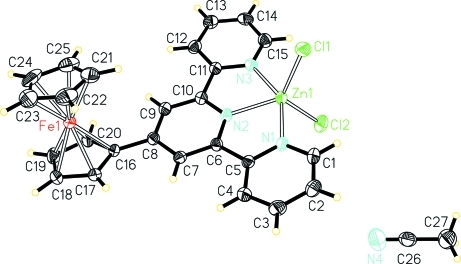
The molecular structure of the title complex, with displacement ellipsoids drawn at the 30% probability level, and H atoms as spheres of arbitrary radius.

## supplementary crystallographic information

### Comment

4'-Ferrocenyl-2,2':6',2"-terpyridine (fctpy) has rencently been paid more attentions for its good chelating abilities towards transition metal ions, such as Au^I^, Ru^II^, Co^II^, Fe^II^, Cu^II^, and Zn^II^complexes (Aguado *et al.*, 2005; Constable *et al.*, 1994; Farlow *et al.*, 1993; Tang & Kuang, 2007; Tang & Kuang *et al.*, 2009).some of its complexes exhibited interesting electrochemical and luminescent properties. In this work, a new Zn^II^ complex of fctpy is here reported.

In the title complex, the Zn^II^ atom is five-coordinated by three N atoms from fctpy ligand and two Cl atoms, displaying a trigonal bipyramidal geometry with two N atoms of outer pyridine rings of fctpy ligand located at apical sites. The Zn—N and Zn—Cl bond lengths are in the range of 2.093 (3)—2.210 (4) and 2.2490 (13)—2.2672 (14) Å, respectively. The angles subtended by the terpyridyl ligand are 74.85 (13) and 74.46 (14) °.

### Experimental

The ligand fctpy was synthesized according to the reported procedure (Farlow *et al.*, 1993). A solution of zinc chloride (6.8 mg, 0.05 mmol) and fctpy (21.0 mg, 0.05 mmol) in methanol (10 ml) was stirred for 4 h. The product was filtered off and dried. The product was filtered off and dried. The precipitate were recrystallized from acetonitrile (5 ml) to give red prism-shaped crystals of the title complex after one week. Yield: 16 mg (53.9%).

### Refinement

All H-atoms were positioned geometrically and refined using a riding model with d(C—H) = 0.93 Å, *U*_iso_=1.2*U*_eq_ (C) for aromatic; 0.98 Å, *U*_iso_= 1.2*U*_eq_ (C) for cyclopentadiene; 0.96 Å, *U*_iso_= 1.5*U*_eq_ (C) for CH_3_ atoms.

### Crystal data

**Table:**

[FeZn(C_5_H_5_)Cl_2_(C_20_H_14_N_3_)]·C_2_H_3_N	*F*(000) = 1208
*M**_r_* = 594.61	*D*_x_ = 1.559 Mg m^−^^3^
Monoclinic, *P*2_1_/*c*	Mo *K*α radiation, λ = 0.71073 Å
Hall symbol: -P 2ybc	Cell parameters from 1833 reflections
*a* = 13.799 (2) Å	θ = 2.4–21.3°
*b* = 12.998 (2) Å	µ = 1.75 mm^−^^1^
*c* = 14.594 (2) Å	*T* = 295 K
β = 104.514 (4)°	Prism, red
*V* = 2534.0 (6) Å^3^	0.18 × 0.14 × 0.10 mm
*Z* = 4	

### Data collection

**Table:**

Bruker APEX area-detector diffractometer	4938 independent reflections
Radiation source: fine-focus sealed tube	3301 reflections with *I* > 2σ(*I*)
graphite	*R*_int_ = 0.051
φ and ω scans	θ_max_ = 26.0°, θ_min_ = 2.1°
Absorption correction: multi-scan (*SADABS*; Sheldrick, 1996)	*h* = −17→15
*T*_min_ = 0.743, *T*_max_ = 0.844	*k* = −14→16
12737 measured reflections	*l* = −13→18

### Refinement

**Table:**

Refinement on *F*^2^	Primary atom site location: structure-invariant direct methods
Least-squares matrix: full	Secondary atom site location: difference Fourier map
*R*[*F*^2^ > 2σ(*F*^2^)] = 0.050	Hydrogen site location: inferred from neighbouring sites
*wR*(*F*^2^) = 0.131	H-atom parameters constrained
*S* = 1.02	*w* = 1/[σ^2^(*F*_o_^2^) + (0.0571*P*)^2^] where *P* = (*F*_o_^2^ + 2*F*_c_^2^)/3
4938 reflections	(Δ/σ)_max_ < 0.001
317 parameters	Δρ_max_ = 0.77 e Å^−^^3^
0 restraints	Δρ_min_ = −0.40 e Å^−^^3^

### Special details

**Table:**

Geometry. All e.s.d.'s (except the e.s.d. in the dihedral angle between two l.s. planes) are estimated using the full covariance matrix. The cell e.s.d.'s are taken into account individually in the estimation of e.s.d.'s in distances, angles and torsion angles; correlations between e.s.d.'s in cell parameters are only used when they are defined by crystal symmetry. An approximate (isotropic) treatment of cell e.s.d.'s is used for estimating e.s.d.'s involving l.s. planes.
Refinement. Refinement of *F*^2^ against ALL reflections. The weighted *R*-factor *wR* and goodness of fit *S* are based on *F*^2^, conventional *R*-factors *R* are based on *F*, with *F* set to zero for negative *F*^2^. The threshold expression of *F*^2^ > σ(*F*^2^) is used only for calculating *R*-factors(gt) *etc*. and is not relevant to the choice of reflections for refinement. *R*-factors based on *F*^2^ are statistically about twice as large as those based on *F*, and *R*- factors based on ALL data will be even larger.

### Fractional atomic coordinates and isotropic or equivalent isotropic displacement parameters (Å^2^)

**Table:**

	*x*	*y*	*z*	*U*_iso_*/*U*_eq_	
Fe2	0.15322 (5)	−0.18976 (5)	0.06212 (4)	0.0384 (2)	
Zn1	0.27254 (4)	0.38084 (4)	0.02199 (4)	0.04109 (18)	
Cl3	0.15301 (10)	0.44465 (10)	−0.10085 (9)	0.0582 (4)	
Cl4	0.39084 (10)	0.49174 (10)	0.09763 (10)	0.0595 (4)	
N1	0.1865 (3)	0.3817 (3)	0.1303 (3)	0.0410 (9)	
N2	0.2663 (3)	0.2268 (3)	0.0618 (3)	0.0353 (9)	
N3	0.3560 (3)	0.2940 (3)	−0.0636 (3)	0.0408 (9)	
N4	0.4086 (5)	0.6416 (5)	0.4004 (5)	0.116 (2)	
C1	0.1444 (4)	0.4648 (4)	0.1587 (4)	0.0522 (13)	
H1	0.1497	0.5276	0.1298	0.063*	
C2	0.0938 (4)	0.4612 (4)	0.2286 (4)	0.0572 (14)	
H2	0.0648	0.5200	0.2466	0.069*	
C3	0.0872 (4)	0.3681 (4)	0.2710 (4)	0.0558 (14)	
H3	0.0536	0.3637	0.3187	0.067*	
C4	0.1298 (4)	0.2809 (4)	0.2437 (3)	0.0476 (12)	
H4	0.1262	0.2179	0.2728	0.057*	
C5	0.1783 (3)	0.2902 (3)	0.1714 (3)	0.0356 (10)	
C6	0.2218 (3)	0.2022 (3)	0.1314 (3)	0.0347 (10)	
C7	0.2165 (3)	0.1017 (3)	0.1600 (3)	0.0366 (10)	
H7	0.1869	0.0863	0.2090	0.044*	
C8	0.2560 (3)	0.0232 (3)	0.1149 (3)	0.0370 (10)	
C9	0.3017 (3)	0.0500 (3)	0.0432 (3)	0.0421 (11)	
H9	0.3296	−0.0004	0.0125	0.050*	
C10	0.3053 (3)	0.1532 (3)	0.0181 (3)	0.0361 (10)	
C11	0.3525 (3)	0.1907 (3)	−0.0571 (3)	0.0375 (11)	
C12	0.3885 (4)	0.1273 (4)	−0.1163 (3)	0.0476 (12)	
H12	0.3840	0.0562	−0.1116	0.057*	
C13	0.4318 (4)	0.1714 (4)	−0.1834 (3)	0.0499 (13)	
H13	0.4564	0.1299	−0.2243	0.060*	
C14	0.4378 (4)	0.2756 (4)	−0.1888 (3)	0.0507 (13)	
H14	0.4676	0.3066	−0.2323	0.061*	
C15	0.3984 (4)	0.3343 (4)	−0.1278 (3)	0.0495 (13)	
H15	0.4016	0.4056	−0.1318	0.059*	
C16	0.2486 (3)	−0.0851 (3)	0.1418 (3)	0.0370 (11)	
C17	0.1829 (4)	−0.1262 (3)	0.1942 (3)	0.0435 (12)	
H17	0.1358	−0.0868	0.2208	0.052*	
C18	0.1969 (4)	−0.2339 (4)	0.1999 (3)	0.0539 (14)	
H18	0.1604	−0.2818	0.2310	0.065*	
C19	0.2716 (4)	−0.2618 (4)	0.1534 (4)	0.0510 (13)	
H19	0.2959	−0.3315	0.1467	0.061*	
C20	0.3029 (4)	−0.1705 (3)	0.1163 (3)	0.0462 (12)	
H20	0.3532	−0.1661	0.0795	0.055*	
C21	0.0683 (5)	−0.1180 (5)	−0.0538 (4)	0.0713 (15)	
H21	0.0689	−0.0439	−0.0664	0.086*	
C22	0.0069 (5)	−0.1663 (5)	−0.0061 (4)	0.0748 (15)	
H22	−0.0439	−0.1334	0.0203	0.090*	
C23	0.0298 (5)	−0.2713 (5)	−0.0031 (5)	0.0820 (16)	
H23	−0.0024	−0.3259	0.0250	0.098*	
C24	0.1066 (5)	−0.2835 (5)	−0.0504 (4)	0.0818 (17)	
H24	0.1378	−0.3487	−0.0610	0.098*	
C25	0.1287 (5)	−0.1888 (5)	−0.0803 (4)	0.0752 (15)	
H25	0.1790	−0.1744	−0.1156	0.090*	
C26	0.4023 (5)	0.7265 (5)	0.4002 (4)	0.0667 (16)	
C27	0.3915 (5)	0.8365 (5)	0.3999 (5)	0.089 (2)	
H27A	0.3598	0.8594	0.3370	0.133*	
H27B	0.4563	0.8678	0.4206	0.133*	
H27C	0.3511	0.8556	0.4421	0.133*	

### Atomic displacement parameters (Å^2^)

**Table:**

	*U*^11^	*U*^22^	*U*^33^	*U*^12^	*U*^13^	*U*^23^
Fe2	0.0469 (4)	0.0330 (4)	0.0339 (4)	−0.0007 (3)	0.0076 (3)	−0.0003 (3)
Zn1	0.0501 (4)	0.0310 (3)	0.0439 (3)	−0.0017 (2)	0.0149 (3)	0.0001 (2)
Cl3	0.0724 (9)	0.0544 (8)	0.0457 (8)	0.0163 (7)	0.0108 (6)	0.0017 (6)
Cl4	0.0604 (9)	0.0472 (7)	0.0716 (9)	−0.0133 (6)	0.0179 (7)	−0.0126 (7)
N1	0.050 (2)	0.029 (2)	0.047 (2)	−0.0023 (17)	0.0170 (19)	−0.0052 (18)
N2	0.035 (2)	0.0300 (19)	0.042 (2)	0.0019 (16)	0.0121 (17)	−0.0020 (17)
N3	0.046 (2)	0.041 (2)	0.039 (2)	−0.0018 (18)	0.0165 (18)	0.0009 (18)
N4	0.154 (7)	0.072 (4)	0.113 (6)	0.000 (4)	0.018 (5)	0.012 (4)
C1	0.069 (4)	0.033 (3)	0.056 (3)	0.004 (2)	0.018 (3)	−0.004 (2)
C2	0.067 (4)	0.050 (3)	0.059 (4)	0.012 (3)	0.025 (3)	−0.010 (3)
C3	0.064 (4)	0.061 (3)	0.051 (3)	0.010 (3)	0.031 (3)	−0.003 (3)
C4	0.062 (3)	0.042 (3)	0.043 (3)	0.001 (2)	0.020 (3)	0.002 (2)
C5	0.037 (3)	0.035 (2)	0.034 (3)	−0.0047 (19)	0.008 (2)	−0.003 (2)
C6	0.034 (3)	0.032 (2)	0.038 (3)	−0.0028 (19)	0.010 (2)	0.000 (2)
C7	0.038 (3)	0.037 (3)	0.034 (2)	−0.001 (2)	0.008 (2)	−0.002 (2)
C8	0.036 (3)	0.036 (2)	0.037 (3)	−0.006 (2)	0.005 (2)	−0.001 (2)
C9	0.051 (3)	0.034 (2)	0.042 (3)	0.004 (2)	0.015 (2)	−0.003 (2)
C10	0.034 (3)	0.036 (2)	0.039 (3)	0.000 (2)	0.010 (2)	−0.001 (2)
C11	0.040 (3)	0.035 (3)	0.038 (3)	0.001 (2)	0.011 (2)	−0.002 (2)
C12	0.055 (3)	0.046 (3)	0.046 (3)	−0.001 (2)	0.019 (2)	−0.007 (2)
C13	0.050 (3)	0.057 (3)	0.048 (3)	0.007 (2)	0.021 (2)	−0.006 (3)
C14	0.053 (3)	0.067 (4)	0.038 (3)	−0.005 (3)	0.022 (2)	0.004 (3)
C15	0.051 (3)	0.048 (3)	0.051 (3)	−0.002 (2)	0.015 (3)	0.003 (3)
C16	0.046 (3)	0.027 (2)	0.035 (3)	−0.0047 (19)	0.006 (2)	−0.0026 (19)
C17	0.057 (3)	0.040 (3)	0.033 (3)	−0.008 (2)	0.011 (2)	−0.002 (2)
C18	0.079 (4)	0.038 (3)	0.041 (3)	−0.011 (3)	0.009 (3)	0.007 (2)
C19	0.058 (3)	0.030 (3)	0.058 (3)	0.005 (2)	0.001 (3)	0.001 (2)
C20	0.047 (3)	0.035 (3)	0.055 (3)	−0.001 (2)	0.010 (2)	0.002 (2)
C21	0.082 (3)	0.071 (3)	0.048 (3)	0.004 (3)	−0.008 (2)	0.006 (3)
C22	0.061 (3)	0.097 (3)	0.057 (3)	0.008 (3)	−0.004 (2)	−0.008 (3)
C23	0.076 (3)	0.086 (3)	0.065 (3)	−0.030 (3)	−0.018 (3)	0.007 (3)
C24	0.088 (3)	0.073 (3)	0.061 (3)	0.017 (3)	−0.025 (3)	−0.033 (3)
C25	0.079 (3)	0.094 (3)	0.045 (3)	0.003 (3)	0.001 (2)	−0.012 (3)
C26	0.077 (4)	0.073 (4)	0.049 (4)	−0.007 (3)	0.014 (3)	0.005 (3)
C27	0.100 (5)	0.073 (5)	0.083 (5)	−0.015 (4)	0.003 (4)	−0.007 (4)

### Geometric parameters (Å, °)

**Table:**

Fe2—C24	2.017 (5)	C8—C16	1.471 (6)
Fe2—C25	2.020 (6)	C9—C10	1.394 (6)
Fe2—C21	2.028 (5)	C9—H9	0.9300
Fe2—C23	2.029 (5)	C10—C11	1.491 (6)
Fe2—C18	2.031 (5)	C11—C12	1.374 (6)
Fe2—C20	2.034 (5)	C12—C13	1.393 (6)
Fe2—C22	2.038 (6)	C12—H12	0.9300
Fe2—C16	2.041 (4)	C13—C14	1.361 (7)
Fe2—C17	2.042 (5)	C13—H13	0.9300
Fe2—C19	2.056 (5)	C14—C15	1.384 (7)
Zn1—N2	2.093 (3)	C14—H14	0.9300
Zn1—N1	2.202 (4)	C15—H15	0.9300
Zn1—N3	2.210 (4)	C16—C17	1.429 (6)
Zn1—Cl4	2.2490 (13)	C16—C20	1.439 (6)
Zn1—Cl3	2.2672 (14)	C17—C18	1.412 (6)
N1—C1	1.340 (6)	C17—H17	0.9800
N1—C5	1.350 (5)	C18—C19	1.416 (7)
N2—C10	1.335 (5)	C18—H18	0.9800
N2—C6	1.350 (5)	C19—C20	1.416 (6)
N3—C15	1.329 (6)	C19—H19	0.9800
N3—C11	1.348 (5)	C20—H20	0.9800
N4—C26	1.107 (8)	C21—C25	1.361 (8)
C1—C2	1.374 (7)	C21—C22	1.376 (8)
C1—H1	0.9300	C21—H21	0.9800
C2—C3	1.372 (7)	C22—C23	1.399 (8)
C2—H2	0.9300	C22—H22	0.9800
C3—C4	1.381 (6)	C23—C24	1.412 (9)
C3—H3	0.9300	C23—H23	0.9800
C4—C5	1.389 (6)	C24—C25	1.366 (8)
C4—H4	0.9300	C24—H24	0.9800
C5—C6	1.479 (6)	C25—H25	0.9800
C6—C7	1.379 (6)	C26—C27	1.437 (8)
C7—C8	1.397 (6)	C27—H27A	0.9600
C7—H7	0.9300	C27—H27B	0.9600
C8—C9	1.396 (6)	C27—H27C	0.9600
			
C24—Fe2—C25	39.5 (2)	C10—C9—C8	119.3 (4)
C24—Fe2—C21	66.3 (2)	C10—C9—H9	120.3
C25—Fe2—C21	39.3 (2)	C8—C9—H9	120.3
C24—Fe2—C23	40.9 (3)	N2—C10—C9	121.3 (4)
C25—Fe2—C23	67.5 (3)	N2—C10—C11	114.8 (4)
C21—Fe2—C23	66.9 (3)	C9—C10—C11	123.9 (4)
C24—Fe2—C18	126.2 (2)	N3—C11—C12	121.9 (4)
C25—Fe2—C18	162.1 (2)	N3—C11—C10	114.1 (4)
C21—Fe2—C18	157.2 (3)	C12—C11—C10	124.0 (4)
C23—Fe2—C18	108.8 (2)	C11—C12—C13	118.8 (5)
C24—Fe2—C20	118.5 (2)	C11—C12—H12	120.6
C25—Fe2—C20	106.9 (2)	C13—C12—H12	120.6
C21—Fe2—C20	125.9 (2)	C14—C13—C12	119.6 (5)
C23—Fe2—C20	153.9 (2)	C14—C13—H13	120.2
C18—Fe2—C20	68.3 (2)	C12—C13—H13	120.2
C24—Fe2—C22	67.5 (3)	C13—C14—C15	118.1 (5)
C25—Fe2—C22	66.8 (3)	C13—C14—H14	120.9
C21—Fe2—C22	39.6 (2)	C15—C14—H14	120.9
C23—Fe2—C22	40.2 (2)	N3—C15—C14	123.3 (5)
C18—Fe2—C22	122.5 (3)	N3—C15—H15	118.3
C20—Fe2—C22	163.1 (2)	C14—C15—H15	118.3
C24—Fe2—C16	153.5 (3)	C17—C16—C20	106.9 (4)
C25—Fe2—C16	120.0 (2)	C17—C16—C8	126.6 (4)
C21—Fe2—C16	108.9 (2)	C20—C16—C8	126.4 (4)
C23—Fe2—C16	163.9 (3)	C17—C16—Fe2	69.6 (2)
C18—Fe2—C16	68.64 (18)	C20—C16—Fe2	69.0 (2)
C20—Fe2—C16	41.35 (17)	C8—C16—Fe2	124.3 (3)
C22—Fe2—C16	126.7 (2)	C18—C17—C16	107.8 (4)
C24—Fe2—C17	163.9 (3)	C18—C17—Fe2	69.3 (3)
C25—Fe2—C17	155.6 (2)	C16—C17—Fe2	69.5 (3)
C21—Fe2—C17	122.6 (2)	C18—C17—H17	126.1
C23—Fe2—C17	126.9 (3)	C16—C17—H17	126.1
C18—Fe2—C17	40.57 (18)	Fe2—C17—H17	126.1
C20—Fe2—C17	68.8 (2)	C17—C18—C19	109.3 (4)
C22—Fe2—C17	109.9 (2)	C17—C18—Fe2	70.1 (3)
C16—Fe2—C17	40.96 (17)	C19—C18—Fe2	70.7 (3)
C24—Fe2—C19	107.0 (2)	C17—C18—H18	125.3
C25—Fe2—C19	124.8 (2)	C19—C18—H18	125.3
C21—Fe2—C19	161.6 (3)	Fe2—C18—H18	125.3
C23—Fe2—C19	120.2 (2)	C18—C19—C20	107.3 (4)
C18—Fe2—C19	40.5 (2)	C18—C19—Fe2	68.8 (3)
C20—Fe2—C19	40.51 (18)	C20—C19—Fe2	68.9 (3)
C22—Fe2—C19	156.0 (2)	C18—C19—H19	126.4
C16—Fe2—C19	68.93 (18)	C20—C19—H19	126.4
C17—Fe2—C19	68.5 (2)	Fe2—C19—H19	126.4
N2—Zn1—N1	74.85 (13)	C19—C20—C16	108.6 (4)
N2—Zn1—N3	74.46 (14)	C19—C20—Fe2	70.6 (3)
N1—Zn1—N3	149.31 (13)	C16—C20—Fe2	69.6 (3)
N2—Zn1—Cl4	123.51 (10)	C19—C20—H20	125.7
N1—Zn1—Cl4	95.74 (10)	C16—C20—H20	125.7
N3—Zn1—Cl4	100.82 (10)	Fe2—C20—H20	125.7
N2—Zn1—Cl3	119.82 (10)	C25—C21—C22	109.4 (6)
N1—Zn1—Cl3	98.58 (11)	C25—C21—Fe2	70.0 (4)
N3—Zn1—Cl3	96.82 (10)	C22—C21—Fe2	70.6 (3)
Cl4—Zn1—Cl3	116.64 (5)	C25—C21—H21	125.3
C1—N1—C5	118.8 (4)	C22—C21—H21	125.3
C1—N1—Zn1	125.4 (3)	Fe2—C21—H21	125.3
C5—N1—Zn1	115.7 (3)	C21—C22—C23	107.4 (6)
C10—N2—C6	120.2 (4)	C21—C22—Fe2	69.8 (3)
C10—N2—Zn1	120.2 (3)	C23—C22—Fe2	69.6 (3)
C6—N2—Zn1	119.6 (3)	C21—C22—H22	126.3
C15—N3—C11	118.2 (4)	C23—C22—H22	126.3
C15—N3—Zn1	125.5 (3)	Fe2—C22—H22	126.3
C11—N3—Zn1	115.8 (3)	C22—C23—C24	106.5 (6)
N1—C1—C2	122.8 (5)	C22—C23—Fe2	70.2 (3)
N1—C1—H1	118.6	C24—C23—Fe2	69.1 (3)
C2—C1—H1	118.6	C22—C23—H23	126.7
C3—C2—C1	117.9 (5)	C24—C23—H23	126.7
C3—C2—H2	121.0	Fe2—C23—H23	126.7
C1—C2—H2	121.0	C25—C24—C23	108.1 (6)
C2—C3—C4	120.8 (5)	C25—C24—Fe2	70.4 (3)
C2—C3—H3	119.6	C23—C24—Fe2	70.1 (3)
C4—C3—H3	119.6	C25—C24—H24	126.0
C3—C4—C5	118.0 (5)	C23—C24—H24	126.0
C3—C4—H4	121.0	Fe2—C24—H24	126.0
C5—C4—H4	121.0	C21—C25—C24	108.5 (6)
N1—C5—C4	121.6 (4)	C21—C25—Fe2	70.7 (4)
N1—C5—C6	114.8 (4)	C24—C25—Fe2	70.1 (4)
C4—C5—C6	123.6 (4)	C21—C25—H25	125.7
N2—C6—C7	121.4 (4)	C24—C25—H25	125.7
N2—C6—C5	114.8 (4)	Fe2—C25—H25	125.7
C7—C6—C5	123.8 (4)	N4—C26—C27	178.5 (8)
C6—C7—C8	119.5 (4)	C26—C27—H27A	109.5
C6—C7—H7	120.2	C26—C27—H27B	109.5
C8—C7—H7	120.2	H27A—C27—H27B	109.5
C9—C8—C7	118.2 (4)	C26—C27—H27C	109.5
C9—C8—C16	120.9 (4)	H27A—C27—H27C	109.5
C7—C8—C16	120.8 (4)	H27B—C27—H27C	109.5
			
N2—Zn1—N1—C1	−177.1 (4)	C17—Fe2—C18—C19	−120.0 (4)
N3—Zn1—N1—C1	−177.7 (3)	C17—C18—C19—C20	−1.4 (6)
Cl4—Zn1—N1—C1	59.7 (4)	Fe2—C18—C19—C20	58.3 (3)
Cl3—Zn1—N1—C1	−58.4 (4)	C17—C18—C19—Fe2	−59.7 (3)
N2—Zn1—N1—C5	4.2 (3)	C24—Fe2—C19—C18	−126.3 (4)
N3—Zn1—N1—C5	3.6 (5)	C25—Fe2—C19—C18	−166.0 (3)
Cl4—Zn1—N1—C5	−119.0 (3)	C21—Fe2—C19—C18	167.9 (6)
Cl3—Zn1—N1—C5	122.9 (3)	C23—Fe2—C19—C18	−83.8 (4)
N1—Zn1—N2—C10	176.3 (3)	C20—Fe2—C19—C18	119.4 (4)
N3—Zn1—N2—C10	−4.1 (3)	C22—Fe2—C19—C18	−53.6 (7)
Cl4—Zn1—N2—C10	−96.9 (3)	C16—Fe2—C19—C18	81.4 (3)
Cl3—Zn1—N2—C10	85.0 (3)	C17—Fe2—C19—C18	37.3 (3)
N1—Zn1—N2—C6	−3.1 (3)	C24—Fe2—C19—C20	114.2 (4)
N3—Zn1—N2—C6	176.6 (3)	C25—Fe2—C19—C20	74.5 (4)
Cl4—Zn1—N2—C6	83.8 (3)	C21—Fe2—C19—C20	48.5 (8)
Cl3—Zn1—N2—C6	−94.4 (3)	C23—Fe2—C19—C20	156.7 (4)
N2—Zn1—N3—C15	178.7 (4)	C18—Fe2—C19—C20	−119.4 (4)
N1—Zn1—N3—C15	179.3 (3)	C22—Fe2—C19—C20	−173.1 (5)
Cl4—Zn1—N3—C15	−59.3 (4)	C16—Fe2—C19—C20	−38.1 (3)
Cl3—Zn1—N3—C15	59.6 (4)	C17—Fe2—C19—C20	−82.2 (3)
N2—Zn1—N3—C11	7.0 (3)	C18—C19—C20—C16	1.2 (6)
N1—Zn1—N3—C11	7.6 (5)	Fe2—C19—C20—C16	59.4 (3)
Cl4—Zn1—N3—C11	129.0 (3)	C18—C19—C20—Fe2	−58.3 (3)
Cl3—Zn1—N3—C11	−112.1 (3)	C17—C16—C20—C19	−0.6 (5)
C5—N1—C1—C2	0.5 (7)	C8—C16—C20—C19	−177.9 (4)
Zn1—N1—C1—C2	−178.2 (4)	Fe2—C16—C20—C19	−60.1 (3)
N1—C1—C2—C3	0.5 (8)	C17—C16—C20—Fe2	59.5 (3)
C1—C2—C3—C4	−0.3 (8)	C8—C16—C20—Fe2	−117.9 (4)
C2—C3—C4—C5	−0.8 (8)	C24—Fe2—C20—C19	−82.9 (4)
C1—N1—C5—C4	−1.7 (7)	C25—Fe2—C20—C19	−124.2 (3)
Zn1—N1—C5—C4	177.1 (3)	C21—Fe2—C20—C19	−163.1 (3)
C1—N1—C5—C6	176.5 (4)	C23—Fe2—C20—C19	−51.0 (7)
Zn1—N1—C5—C6	−4.7 (5)	C18—Fe2—C20—C19	37.5 (3)
C3—C4—C5—N1	1.8 (7)	C22—Fe2—C20—C19	170.3 (7)
C3—C4—C5—C6	−176.2 (4)	C16—Fe2—C20—C19	119.5 (4)
C10—N2—C6—C7	1.0 (6)	C17—Fe2—C20—C19	81.3 (3)
Zn1—N2—C6—C7	−179.7 (3)	C24—Fe2—C20—C16	157.6 (3)
C10—N2—C6—C5	−177.7 (4)	C25—Fe2—C20—C16	116.4 (3)
Zn1—N2—C6—C5	1.6 (5)	C21—Fe2—C20—C16	77.5 (4)
N1—C5—C6—N2	2.2 (6)	C23—Fe2—C20—C16	−170.4 (5)
C4—C5—C6—N2	−179.7 (4)	C18—Fe2—C20—C16	−81.9 (3)
N1—C5—C6—C7	−176.5 (4)	C22—Fe2—C20—C16	50.8 (9)
C4—C5—C6—C7	1.7 (7)	C17—Fe2—C20—C16	−38.2 (3)
N2—C6—C7—C8	−1.5 (7)	C19—Fe2—C20—C16	−119.5 (4)
C5—C6—C7—C8	177.1 (4)	C24—Fe2—C21—C25	−37.4 (4)
C6—C7—C8—C9	1.4 (6)	C23—Fe2—C21—C25	−82.1 (4)
C6—C7—C8—C16	−178.0 (4)	C18—Fe2—C21—C25	−165.8 (5)
C7—C8—C9—C10	−0.9 (6)	C20—Fe2—C21—C25	71.6 (5)
C16—C8—C9—C10	178.5 (4)	C22—Fe2—C21—C25	−120.2 (6)
C6—N2—C10—C9	−0.5 (6)	C16—Fe2—C21—C25	114.6 (4)
Zn1—N2—C10—C9	−179.8 (3)	C17—Fe2—C21—C25	157.8 (4)
C6—N2—C10—C11	−179.8 (4)	C19—Fe2—C21—C25	34.7 (9)
Zn1—N2—C10—C11	0.8 (5)	C24—Fe2—C21—C22	82.8 (4)
C8—C9—C10—N2	0.5 (7)	C25—Fe2—C21—C22	120.2 (6)
C8—C9—C10—C11	179.8 (4)	C23—Fe2—C21—C22	38.1 (4)
C15—N3—C11—C12	−1.9 (7)	C18—Fe2—C21—C22	−45.6 (7)
Zn1—N3—C11—C12	170.4 (3)	C20—Fe2—C21—C22	−168.2 (3)
C15—N3—C11—C10	178.9 (4)	C16—Fe2—C21—C22	−125.2 (4)
Zn1—N3—C11—C10	−8.7 (5)	C17—Fe2—C21—C22	−82.0 (4)
N2—C10—C11—N3	5.4 (6)	C19—Fe2—C21—C22	154.9 (6)
C9—C10—C11—N3	−173.9 (4)	C25—C21—C22—C23	−0.2 (7)
N2—C10—C11—C12	−173.7 (4)	Fe2—C21—C22—C23	−59.6 (4)
C9—C10—C11—C12	7.0 (7)	C25—C21—C22—Fe2	59.5 (4)
N3—C11—C12—C13	1.5 (7)	C24—Fe2—C22—C21	−79.6 (4)
C10—C11—C12—C13	−179.5 (4)	C25—Fe2—C22—C21	−36.5 (4)
C11—C12—C13—C14	0.2 (7)	C23—Fe2—C22—C21	−118.5 (6)
C12—C13—C14—C15	−1.3 (7)	C18—Fe2—C22—C21	160.8 (3)
C11—N3—C15—C14	0.8 (7)	C20—Fe2—C22—C21	34.8 (10)
Zn1—N3—C15—C14	−170.7 (4)	C16—Fe2—C22—C21	74.5 (4)
C13—C14—C15—N3	0.8 (8)	C17—Fe2—C22—C21	117.5 (4)
C9—C8—C16—C17	−162.3 (4)	C19—Fe2—C22—C21	−160.8 (5)
C7—C8—C16—C17	17.1 (7)	C24—Fe2—C22—C23	38.9 (4)
C9—C8—C16—C20	14.5 (7)	C25—Fe2—C22—C23	82.0 (4)
C7—C8—C16—C20	−166.1 (4)	C21—Fe2—C22—C23	118.5 (6)
C9—C8—C16—Fe2	−73.5 (5)	C18—Fe2—C22—C23	−80.7 (5)
C7—C8—C16—Fe2	105.9 (5)	C20—Fe2—C22—C23	153.3 (7)
C24—Fe2—C16—C17	−167.0 (5)	C16—Fe2—C22—C23	−167.0 (4)
C25—Fe2—C16—C17	160.0 (3)	C17—Fe2—C22—C23	−124.0 (4)
C21—Fe2—C16—C17	118.3 (3)	C19—Fe2—C22—C23	−42.3 (8)
C23—Fe2—C16—C17	46.4 (9)	C21—C22—C23—C24	0.0 (6)
C18—Fe2—C16—C17	−37.5 (3)	Fe2—C22—C23—C24	−59.8 (4)
C20—Fe2—C16—C17	−118.4 (4)	C21—C22—C23—Fe2	59.8 (4)
C22—Fe2—C16—C17	77.9 (4)	C24—Fe2—C23—C22	−117.5 (6)
C19—Fe2—C16—C17	−81.1 (3)	C25—Fe2—C23—C22	−80.2 (4)
C24—Fe2—C16—C20	−48.6 (6)	C21—Fe2—C23—C22	−37.5 (4)
C25—Fe2—C16—C20	−81.6 (3)	C18—Fe2—C23—C22	118.5 (4)
C21—Fe2—C16—C20	−123.3 (3)	C20—Fe2—C23—C22	−162.8 (5)
C23—Fe2—C16—C20	164.8 (7)	C16—Fe2—C23—C22	40.5 (10)
C18—Fe2—C16—C20	80.9 (3)	C17—Fe2—C23—C22	76.9 (5)
C22—Fe2—C16—C20	−163.7 (3)	C19—Fe2—C23—C22	161.5 (4)
C17—Fe2—C16—C20	118.4 (4)	C25—Fe2—C23—C24	37.2 (4)
C19—Fe2—C16—C20	37.3 (3)	C21—Fe2—C23—C24	80.0 (4)
C24—Fe2—C16—C8	71.9 (6)	C18—Fe2—C23—C24	−124.1 (4)
C25—Fe2—C16—C8	38.9 (5)	C20—Fe2—C23—C24	−45.3 (7)
C21—Fe2—C16—C8	−2.7 (5)	C22—Fe2—C23—C24	117.5 (6)
C23—Fe2—C16—C8	−74.7 (9)	C16—Fe2—C23—C24	158.0 (7)
C18—Fe2—C16—C8	−158.5 (4)	C17—Fe2—C23—C24	−165.7 (4)
C20—Fe2—C16—C8	120.6 (5)	C19—Fe2—C23—C24	−81.0 (4)
C22—Fe2—C16—C8	−43.2 (5)	C22—C23—C24—C25	0.2 (7)
C17—Fe2—C16—C8	−121.1 (5)	Fe2—C23—C24—C25	−60.4 (4)
C19—Fe2—C16—C8	157.9 (4)	C22—C23—C24—Fe2	60.6 (4)
C20—C16—C17—C18	−0.3 (5)	C21—Fe2—C24—C25	37.2 (4)
C8—C16—C17—C18	177.1 (4)	C23—Fe2—C24—C25	118.7 (6)
Fe2—C16—C17—C18	58.9 (3)	C18—Fe2—C24—C25	−165.0 (4)
C20—C16—C17—Fe2	−59.2 (3)	C20—Fe2—C24—C25	−82.1 (4)
C8—C16—C17—Fe2	118.2 (4)	C22—Fe2—C24—C25	80.3 (4)
C24—Fe2—C17—C18	39.4 (9)	C16—Fe2—C24—C25	−47.8 (7)
C25—Fe2—C17—C18	−165.3 (5)	C17—Fe2—C24—C25	164.3 (7)
C21—Fe2—C17—C18	159.3 (3)	C19—Fe2—C24—C25	−124.5 (4)
C23—Fe2—C17—C18	75.1 (4)	C25—Fe2—C24—C23	−118.7 (6)
C20—Fe2—C17—C18	−80.9 (3)	C21—Fe2—C24—C23	−81.5 (4)
C22—Fe2—C17—C18	117.1 (3)	C18—Fe2—C24—C23	76.4 (5)
C16—Fe2—C17—C18	−119.4 (4)	C20—Fe2—C24—C23	159.2 (4)
C19—Fe2—C17—C18	−37.2 (3)	C22—Fe2—C24—C23	−38.3 (4)
C24—Fe2—C17—C16	158.8 (8)	C16—Fe2—C24—C23	−166.5 (4)
C25—Fe2—C17—C16	−45.9 (7)	C17—Fe2—C24—C23	45.6 (10)
C21—Fe2—C17—C16	−81.3 (4)	C19—Fe2—C24—C23	116.8 (4)
C23—Fe2—C17—C16	−165.4 (3)	C22—C21—C25—C24	0.3 (7)
C18—Fe2—C17—C16	119.4 (4)	Fe2—C21—C25—C24	60.1 (4)
C20—Fe2—C17—C16	38.6 (3)	C22—C21—C25—Fe2	−59.8 (4)
C22—Fe2—C17—C16	−123.4 (3)	C23—C24—C25—C21	−0.3 (7)
C19—Fe2—C17—C16	82.2 (3)	Fe2—C24—C25—C21	−60.5 (4)
C16—C17—C18—C19	1.1 (5)	C23—C24—C25—Fe2	60.2 (4)
Fe2—C17—C18—C19	60.0 (3)	C24—Fe2—C25—C21	119.0 (6)
C16—C17—C18—Fe2	−59.0 (3)	C23—Fe2—C25—C21	80.6 (4)
C24—Fe2—C18—C17	−167.4 (3)	C18—Fe2—C25—C21	162.0 (7)
C25—Fe2—C18—C17	160.1 (7)	C20—Fe2—C25—C21	−126.5 (4)
C21—Fe2—C18—C17	−50.3 (7)	C22—Fe2—C25—C21	36.8 (4)
C23—Fe2—C18—C17	−125.2 (3)	C16—Fe2—C25—C21	−83.4 (4)
C20—Fe2—C18—C17	82.4 (3)	C17—Fe2—C25—C21	−50.5 (7)
C22—Fe2—C18—C17	−82.9 (4)	C19—Fe2—C25—C21	−167.4 (4)
C16—Fe2—C18—C17	37.8 (3)	C21—Fe2—C25—C24	−119.0 (6)
C19—Fe2—C18—C17	120.0 (4)	C23—Fe2—C25—C24	−38.4 (4)
C24—Fe2—C18—C19	72.6 (4)	C18—Fe2—C25—C24	43.0 (10)
C25—Fe2—C18—C19	40.1 (9)	C20—Fe2—C25—C24	114.5 (4)
C21—Fe2—C18—C19	−170.2 (5)	C22—Fe2—C25—C24	−82.2 (4)
C23—Fe2—C18—C19	114.8 (3)	C16—Fe2—C25—C24	157.6 (4)
C20—Fe2—C18—C19	−37.5 (3)	C17—Fe2—C25—C24	−169.5 (5)
C22—Fe2—C18—C19	157.1 (3)	C19—Fe2—C25—C24	73.6 (5)
C16—Fe2—C18—C19	−82.1 (3)		
